# Staphylococcal Enterotoxins: Description and Importance in Food

**DOI:** 10.3390/pathogens13080676

**Published:** 2024-08-09

**Authors:** Mirian Yuliza Rubio Cieza, Erika Carolina Romão Bonsaglia, Vera Lucia Mores Rall, Marcos Veiga dos Santos, Nathália Cristina Cirone Silva

**Affiliations:** 1 Department of Food Science and Nutrition, School of Food Engineering, University of Campinas (UNICAMP), Campinas 13083-862, Brazil; mirianrubio1508@gmail.com; 2 Department of Animal Nutrition and Production, School of Veterinary Medicine and Animal Sciences, University of São Paulo (USP), Pirassununga 13635-900, Brazil; erikabonsaglia@gmail.com (E.C.R.B.); mveiga@usp.br (M.V.d.S.); 3 Department of Chemical and Biological Sciences, Institute of Biosciences, Sao Paulo State University, Botucatu 18618-691, Brazil; vera.rall@unesp.br

**Keywords:** *Staphylococcus aureus*, enterotoxins, genes, staphylococcal food poisoning

## Abstract

*Staphylococcus aureus* stands out as one of the most virulent pathogens in the genus *Staphylococcus*. This characteristic is due to its ability to produce a wide variety of staphylococcal enterotoxins (SEs) and exotoxins, which in turn can cause staphylococcal food poisoning (SFP), clinical syndromes such as skin infections, inflammation, pneumonia, and sepsis, in addition to being associated with the development of inflammation in the mammary glands of dairy cattle, which results in chronic mastitis and cell necrosis. SEs are small globular proteins that combine superantigenic and emetic activities; they are resistant to heat, low temperatures, and proteolytic enzymes and are tolerant to a wide pH range. More than 24 SE genes have been well described (SEA-SEE, SEG, SEH, SEI, SEJ, SElK, SElL, SElM, SElN, SElO, SElP, SElQ, SElR, SElS, SElT, SElU, SElV, SElW, SElX, SElY, and SElZ), being a part of different SFP outbreaks, clinical cases, and isolated animal strains. In recent years, new genes (*sel26*, *sel27*, *sel28*, *sel31*, *sel32*, and *sel33*) from SEs have been described, as well as two variants (*seh-2p* and *ses-3p*) resulting in a total of thirty-three genes from Ses, including the nine variants that are still in the process of genetic and molecular structure evaluation. SEs are encoded by genes that are located in mobile genetic elements, such as plasmids, prophages, pathogenicity islands, and the enterotoxin gene cluster (*egc*), and housed in the genomic island of *S. aureus*. Both classical SEs and SE-like toxins (SEls) share phylogenetic relationships, structure, function, and sequence homology, which are characteristics for the production of new SEs through recombination processes. Due to the epidemiological importance of SEs, their rapid assessment and detection have been crucial for food security and public health; for this reason, different methods of identification of SEs have been developed, such as liquid chromatography coupled with high-resolution mass spectrometry (LC-HRMS), molecular methods, and whole-genome sequencing; providing the diagnosis of SEs and a better understanding of the occurrence, spread, and eradication of SEs. This review provides scientific information on the enterotoxins produced by *S. aureus*, such as structural characteristics, genetic organization, regulatory mechanisms, superantigen activity, mechanisms of action used by SEs at the time of interaction with the immune system, methods of detection of SEs, and recent biocontrol techniques used in food.

## 1. Introduction

*Staphylococcus aureus* is the most virulent species of the *Staphylococcus* genus and is the most isolated pathogen in clinical cases [1], SFP cases, dairy cattle with mastitis, and dairy products. It causes a wide range of infectious diseases in humans, in addition to SFP, which results from the ingestion of preformed SEs in food contaminated by strains of *S. aureus* [2]. Reports of SFP cases involving *S. aureus* as the primary agent have been documented for decades [2]. In 1930, the first case of SFP was reported in eleven people who presented symptoms such as vomiting and abdominal pain after eating cake filled with cream contaminated with yellow hemolytic *Staphylococcus* [3]. In France, *S. aureus* is reported as the second cause of SFP after evaluating strains from 31 outbreaks of SFP between 1981 and 2002 [4]. Since then, different cases of SFP have been reported from different regions of the world, such as Japan, which has recorded 13,420 cases of SFP due to dairy consumption [5]; in Switzerland 2014 reported cases of SFP in a school due to the consumption of cheese made from raw milk [6]; the United States [2], up to the most recent case reported in Hangzhou, southeast China [7], where *S. aureus* is the main pathogen responsible and cause of SFP. Therefore, *S. aureus* is considered an enterotoxigenic pathogen because of its ability to produce toxins that trigger several diseases, including SFP and toxic shock syndrome [8]. 

Enterotoxigenic *S. aureus* has been identified in various types of foods of animal origin, such as cheese, raw milk, cooked meat, sheep milk, chicken, canned meat, and tuna, among others. It has also been identified in ready-for-consumption food products, such as bowls, pancakes, spaghetti, creams, savory sushi, and cereals such as rice [4,9]. Furthermore, it has also been isolated from containers used in food preparation [7], making food and food products one of the most common vehicles of *S. aureus* transmission.

The main source of food contamination stems from human carriers of microorganisms, which then contaminate food through improper handling practices or via respiratory secretions during coughing and sneezing. Conversely, contamination of animal origin, particularly through derived products such as red meat [9] and dairy items, occurs from animals infected with conditions such as mastitis, which represents a significant potential source of contamination in dairy products [7,9]. These two sources of contamination have been identified as the primary causes of most SFP cases. Typical symptoms of SFP are abdominal pain, vomiting, diarrhea, and fever, and the incubation period varies from 2.5 h (children < 10 years) to 7 h (adults), with concentration levels between >6 and >200 ng of SE/g of food [6].

## 2. *Staphylococcus aureus* Enterotoxins

The virulence of *S. aureus* is mediated by a wide variety of SEs and exotoxins, which are the main cause of clinical syndromes and SFP. SEs are extracellular proteins [9] resistant to heat, cold, and proteolytic enzymes [10]. They can exert emetic and superantigen activity in humans after consumption of contaminated foods [11], with symptoms usually appearing between 30 min and 8 h [12].

More than 24 SE genes have been identified from different outbreaks of SFP, clinical cases and strains isolated from animals, most of which have been well described (SEA-SEE, SEG, SEH, SEI, SElJ, SElK, SElL, SElM, SElN, SElO, SElP, SElQ, SElR, SElS, SElT, SElU, SElV, SElW, SElX, SElY, and SElZ) [13]. The first five SEs identified are named in the order in which they were discovered (SEA, SEB, SEC, SED, and SEE); they are also known as the classic enterotoxins as they are the SEs commonly associated with outbreaks of intoxication because of their ability to induce emesis in humans [14]. The other enterotoxins classified as SEs are similar to the classic enterotoxins (SEls) and are also considered a significant threat to humans. These SEIs have been identified in cases of SFP even without the presence of classic SEs, as reported by Umeda et al. [15]. However, both classic SEs and SEls share phylogenetic relationships, structure, function, and sequence homology [16], which are characteristics conducive to the production of new SEs through recombination processes [17].

In recent years, additional new genes encoding SEs (*sel26*, *sel27*, *sel28*, *sel29*, *sel30*, *sel31*, *sel32*, and *sel33*) have been identified, in addition to two variants (*ses-2p* and *ses-3p*). They are present in data from 133 *S. aureus* genomes collected between 1924 and 2016 [18]. In addition, Aung et al. [18] reported the presence of *selw*, *selx*, *selz*, *sel26*, and *sel27*, in methicillin-resistant clinical isolates. A total of 33 SE genes were identified, including the nine variants currently undergoing epidemiological evaluation and characterization [17].

## 3. Origin and Identification of Classical Staphylococcal Enterotoxins (A-SEA, B-SEB), C-SEC, D-SED and E-SEE) and SEls

From 1953 to 1960, more than one immunological type of enterotoxin was reported as the cause of SFP incidents (cooked ham) and enteritis (the case of a child with acute nonspecific diarrhea); the types were initially identified as enterotoxin F (food poisoning) and E (enteritis). However, in 1962, they were designated as enterotoxin A and B, respectively, by the American Society for Microbiology in Kansas City [19].

Enterotoxin C was identified in 1965 and was the product of an investigation into two staphylococcal strains, 137 (isolated from an abscess on the leg of a patient) and 361 (isolated from cooked chicken from a case of SPF in 1962). Both SEs were compared with enterotoxins A and B and later identified to be antigenically different. It was then formally recognized as enterotoxin C, with its prototype strain 137 (ATCC19095), by the Food and Drug Administration in Washington, the National Department of Health and Drugs in Ottawa, Canada, and by the Health Education and Wellness Center of the United States [20]. SEC were characterized as proteins with a molecular weight of 34,000 and composed of 296 (strain 137) and 294 (361) amino acids [21]. The toxicity potential of SEC was demonstrated in monkeys, where intragastric administration of 5 µg of enterotoxin led to the production of emesis between 2 and 5 h after [22]. That same year, strains of *S. aureus* producing SEC were identified in humans, food, and animals, demonstrating that strains isolated from humans showed greater SEC production, in addition to the different sequences that facilitated survival in their hosts [21].

SED was identified in 1967 from strain 494 (ATCC 23235), isolated from turkey salad waste. It was epidemiologically responsible for SFP in 150 children in Washington [23]. Initially classified as a simple protein with a single polypeptide chain, composed of 236 amino acids and stable in the pH range of 1.2–10.7 [24]. Its toxicity was evaluated in monkeys, inducing 100% emesis after being administered 1.25 µg/kg [25]. Enterotoxin E was identified from the FRI-326 strain, originally isolated in 1960 from an outbreak of SFP [26].

SEH was Identified from *S. aureus* FRI-569 and classified as a single-chain protein with amino acid sequences distinctive from A, B, C, D, and E, and a toxic shock toxin [27]. SEG and SEI were identified from enterotoxigenic strains FRI (Food Research Institute, University of Wisconsin—Madison). They were classified as emetic toxins of 777 and 229 nucleotides that encode proteins with 258 (with 39% similarity between the amino acids of SEB and SEC) and 242 (with 26 to 28% similarity to SEA, SEE, and SED) amino acids, respectively [28]. Since then, SEls have been discovered as part of isolates from foods, clinical cases, and animals. SEls share phylogenetic relationships, structure, function, and sequence homology with SE and are considered products of genetic recombination processes.

## 4. Genetic Organization of SEs and SEls

SEs are globular proteins that make up a family of superantigens (SAgs) with molecular weights between 19 and 29 KD [29]. They are encoded by genes that are located on mobile genetic elements (Table 1), such as plasmids, prophages, pathogenicity islands [30], and the enterotoxin gene clusters (*egc*) [31], housed on the *S. aureus* genomic island [17].

The locations of enterotoxin genes vary extensively. These genes can be carried by plasmids (*seb*, *sed*, *selj*, *selr*, *sels*, and *selt*), phages (*sea* and *see*), or genomic islands (*seb*, *sec*, *seg*, *seh*, *sei*, *selk*, *sel*, *selm*, *seln*, *selo*, *selt*, and *selq*), depending on the isolate’s origin [32]. For instance, the gene *sea*, encoding SEA, was identified in the prophage region φMu3A of *S. aureus* and is also present in the φSA3 region alongside other genes (*sea*, *selk*, and *selq*) in different strains. The *seb* gene was identified on a pathogenicity island (SAPlIvm60), while *sed*, *selr*, and *selj* were found in the plB485-like plasmid. The *seh* gene was located in the φSa3ms region, with the *selo* gene adjacent to it [33]. Furthermore, the gene encoding *sec* is situated on either a plasmid or a pathogenicity island [10].

Regarding the genes encoding toxins SElJ, SElR, SES, and SElT, they are located on plasmids in various combinations. For example, SED/SElJ is situated in pIB485, SEQ/SEK/SEB is located in SaPI, and SEC/SElL is found in SaPIbov [29]; furthermore, the latter two are found together in the same genome, according to studies conducted by [34]. Conversely, genes encoding SEls SElW and SElX are located on the chromosome [33].

The *egc* cluster houses the genes (*seg*, *sei*, *selm*, *seln*, *selo*, and *selu*) [7] that encode enterotoxins SEG, SEI, SElK, SElL, SElM, SElN, and SElO [29,31]. The identification of one of these genes implies the presence of another being linked to each other but without the presence of the classic ES genes [7]. The *egc* cluster is divided into types, *egc1* (*seg*, *selm*, *ψent1*–*ψent2*, *sei*, *selm*, and *seo*) located in a vSaβ type I, *egc5* (*seg*, *selm*, *selu2*, *sei*, *selm*, and *selo*) present in the vSaβ type I and vSaβ type IV pathogenicity island [33].

The *egc* cluster has already been found in high prevalence from clinical isolates [35] and animal hosts of *S. aureus* strains [36]. More recently, it has been reported in strains isolated from foods, mainly in cheeses made from raw milk and dairy products from Brazil and Belgium [31,37]; furthermore, they have already been reported as part of an SPF outbreak in Hangzhou, China [7].

**Table 1:** Characteristics of staphylococcal enterotoxins produced by *S. aureus* strains: genetic locations, superantigen and emetic activity, and identification.

SEs and SEls	Genetic Element	Superantigenic Activity (SA)/Emetic Activity (EA)	Identification
SEA	Prophage	SA^+^/EA^+^	SFP, Bovine, Caprine, Ovine, Dairy products, Human
SEB	Chromosome, SaPI3, Plasmid (pZA10)	SA^+^/EA^+^	SFP, Bovine, Caprine, Ovine, Dairy products, Human
SEC	SaPI	SA^+^/EA^+^	SFP, Bovine, Caprine, Ovine, Dairy products, Human
SEC-1	SaPI	SA^+^/EA^+^	Human
SEC-2	SaPI	SA^+^/NE	Human
SEC-3	SaPI	SA^+^/EA^+^	Human
SEC_-bovine_	SaPIbov		Specific to bovine
SEC_-ovine_	SaPIov		Specific to ovine
SED	Plasmid (pIB485)	SA^+^/EA^+^	SFP, Bovine
SEE	Prophage (Hypothetical location)	SA^+^/EA^+^	Unpasteurized milk soft cheese, SFP
SEG	*egc1*, *egc2*, *egc3*, *egc4*	SA^+^/EA^+^	Bovine
SEH	Transposon (MGEmw2/mssa476 *seh*/Δ*seo*)	SA^+^/EA^+^	Empyema human
SElI	*egc1*, *egc2*, *egc3*	SA^+^/EA^+^	Mastitis cows, humans
SElJ	Plasmid (pIB485, pF5)	SA^+^/NE	Ham (epidemiologically implicated in food poisoning)
SElK	Prophages, SaPI1, SaPI3, SaPI5, SaPIbov1	SA^+^/NE	Human
SElL	SaPIn1, SaPIm1, SaPImw2, SaPIbov1	SA^+^/EA^+^	Human
SElM	*egc1*, *egc2*	SA^+^/EA^+^	Bovine
SElN	*egc1*, *egc2*, *egc3*, *egc4*	SA^+^/EA^+^	Human
SElO	*egc1*, *egc2*, *egc3*, *egc4*, Transposon	SA^+^/EA^+^	Human
SElP	Prophage (Sa3n)	SA^+^/EA^+^	Human, Ulcer
SElQ	SaPI1, SaPI3, SaPI5, Prophage	SA^+^/EA^+^	Human
SElR	Plasmid (pIB485, pF5)	SA^+^/EA^+^	Human
SElS	Plasmid (pF5)	SA^+^/EA^+^	*
SElT	Plasmid (pF5)	SA^+^/EA^+^	*
SElU	*egc2*, *egc3*	SA^+^/NE	Human
SElV	*egc4*	SA^+^/NE	*
SElW	*egc4*	SA^+^/NE	Human
SElX	Chromosome	SA^+^/NE	Human, Raw Meat, milk
SElY	Chromosome	SA^+^/NE	Human
SElZ	Chromosome	NE/NE	Bovine
*sel26* **	Chromosome	NE/NE	Sheep, human
*sel27* **	SaPI	NE/NE	Derived from NCTC 5663
*sel28* **	ilha genômica νSaβ	NE/NE	Derived from NCTC 5663
*sel29* **	*	NE/NE	Human
*sel30* **	Plasmid ***	NE/NE	*
*sel31* **	Plasmid ***	NE/NE	Human
*sel32* **	Plasmid ***	NE/NE	
*sel33* **	Plasmid ***	NE/NE	

Positive reaction (SA^+^), Not found(*), Not evaluated (NE), Recently discovered (**) enterotoxin genes (nomenclature or description of them is not yet clear, in addition to studies of staphylococcal protein production; these have been placed in the table to show their existence for future research), and probable origin (***). All information was collected from the following authors [7,14,17,38].

## 5. Structural Characteristics of SEs and SEls

SEs and SEls are globular proteins with molecular weights of 19 to 29 kDa and are structurally homologous with similar nucleotide and amino acid sequences; according to these characteristics, they are classified by groups: group I (SelX and SElY), have primary amino acid sequences, do not have the cystine emetic loop, and contains a low-density major histocompatibility class (MHC) II binding site in its O/B folds that interacts with the α-chains of MHC II molecules [38,39,40,41]. Group II (SEB, SEC, SEG, SElU, and SElW) contains the structure of SAg, with a cystine loop that has a sequence of 10 to 19 amino acids; it contains an MHC II site, the MHC II α-strand binding site, and the Vβ-TCR (T-cell receptor) binding site, and is located at the top front of the molecules [42,43,44,45].

**Figure 1:**
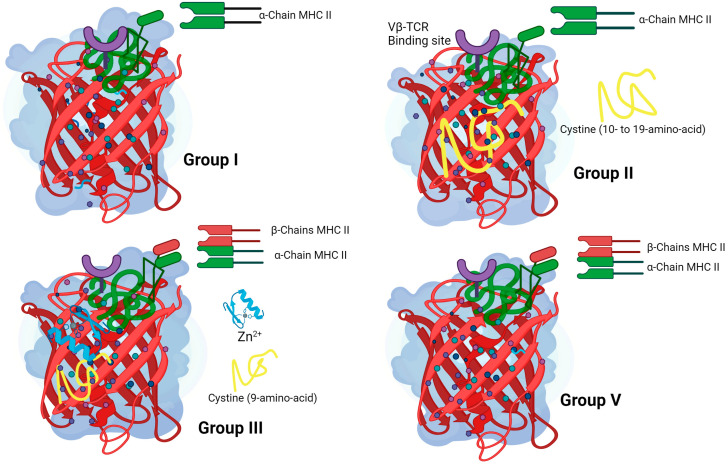
Structural characteristics of SEs and Sels: group I (SElX, SElY), group II (SEB, SEC, SEG, SElU, and SElW), group III (SEA, SED, SEE, SEH, SElJ, SElN, and SElP), and group V (SEI, SElK, SElL, SElM, SElQ, SElT, and SElV). Group I lacks the cystine emetic loop and contains only one low-density major histocompatibility class (MHC) II binding site; group II contains a cystine loop with a sequence of 10 to 19 amino acids, the MHC II site of the α chain, and the Vβ-TCR binding site; group III contains the nine-amino acid cystine loop of the MHC II binding sites (α and β) and the β chain-dependent Zn^2+^ MHC II binding site; group V contains MHC II (α and β) but lacks the cystine loop. Created with BioRender.com.

Group III (SEA, SED, SEE, SEH, SElJ, SElN, and SElP) contains the nine amino acid cystine loop, the low-affinity MHC II α-chain binding site, and the high-affinity Zn^2+^ MHC II link-to-β. In addition, the presence of two MHC II sites in this group allows the SEs to cross the bridge between MHC II molecules in antigen-presenting cells (APCs), which increases the SAg potential, making it 10 to 100 times more active in the production of cytokines by T cells [42,46,47]. Group IV is not produced by *S. aureus*, while group V (SEI, SElK, SElL, SElM, SElQ, SElT, and SElV) of this group contains low-density MHC II but lacks the cystine loop, which is a critical aspect for the specificity of these SAgs with their respective T-cell receptors (Figure 1) [27,48,49]. The newly identified SEl genes have not yet been studied for the production of staphylococcal proteins and are lacking information regarding the structure of protein molecules.

Despite their differences in amino acid sequences, their secondary structures are composed of α-helix and β sheets, and the three-dimensional structures have very similar conformations. However, the canonical structure consists of a domain A and B connected by an α-helix. The A domain contains the amino and the carboxyl-terminal. The interphases between A and B are marked by α-helices, forming a long groove on the back face of the molecule and a shallow cavity at the top. The inner region of the β is hydrophobic, and the outer surface is covered by hydrophilic residues (Figure 1) [38].

## 6. Gene Expression of Enterotoxins and Regulatory Mechanisms

The regulation of gene expression in *S. aureus* is an intricate process that is coordinated by various regulatory factors that form complex networks and is predominantly at the transcriptional level. Over the decades, studies have consistently highlighted the crucial role of the accessory gene regulator (*agr*) as the main regulatory mechanism, and it was identified as the central transcriptional regulator of virulence genes in 1986 [50]. Comprised of chromosomal genes, the *agr* system includes two transcripts, RNAII and RNAIII, controlled by promoters P2 and P3. RNAII is encoded by four genes (*agr*A, *agr*B, *agr*C, and *agr*D), each with distinct functions: *agr*A is a regulatory protein, *agr*C is a histidine kinase, *agr*B is a transport protein, and *agr*D is an autoinducing peptide. The RNAIII transcript, encoded by the hld gene, regulates the agr system’s and virulence genes’ expression [51].

Despite *agr* being the main regulatory mechanism, the expression of genes encoding *S. aureus* virulence factors can also be controlled by other systems. These include positive and negative gene regulation mechanisms involving antisense RNA, transcription factors (such as RNAIII), SarA family proteins, MgrA and SigmaB [52,53], and a toxin-repressing transcription factor (Rot), one of the central regulators of regulatory networks in *S. aureus* virulence [54]. The *agr* system controls some enterotoxins, and the functioning of these regulatory systems depends on factors such as pH, atmosphere, cell growth phase, and bacterial population density [55]. Studies, such as Derzelle et al. [56], have shown four distinct patterns of enterotoxin expression during the cell cycle: (I) a constant abundance of mRNA throughout bacterial growth (*sea*, *see*, *sej*, *selk*, *selq*, and *selp*); (II) a slight decrease in transcription levels (SEG, SEI, SElM, SElN, SElO, SElU); (III) a drastically induced expression at the end of the exponential growth phase *(seb*, *sec*, *seh*); (IV) a moderate post-exponential increase in mRNA levels (<10 times) (*sed*, *ser*, and *sell*). This highlights that all strains containing these genes have the potential to produce enterotoxins and that most of these recently described genes are probably not regulated by the *agr* system, emphasizing the complexity of these mechanisms. The induction of enterotoxins in *S. aureus* involves an intricate web of regulatory factors, requiring in-depth understanding. Although the *agr* system can influence expression, it is crucial to exercise caution to avoid overemphasizing its action.

Previous research, such as that conducted by Tseng & Stewart [50], suggested that the regulation of SED is not exclusively mediated by this system. Interactions with Rot also play a role in the regulation of this gene, as highlighted by Zeaki et al. [57], where *sed* transcription levels in an *agr* mutant were comparable to the wild-type isogenic strain. In addition, the expression of enterotoxins not regulated by the *agr* system can be influenced by other systems, as well as the presence of prophages. For example, for enterotoxins A (SEA), G (SEG), and K (SElK), expression is associated with the activation of a specific prophage [57,58].

## 7. Superantigen Activity of Classical and Non-Classical Enterotoxins

In the late 1960s, Bergdoll and colleagues first described toxin A secreted by *S. aureus*, calling it a bacterial antigen with potent enterotoxigenic properties [19]. This framework was crucial for the subsequent understanding of staphylococcal enterotoxins associated with food poisoning. In the 1980s, the term “superantigen” was introduced, representing a redefinition in the field of immunology and encompassing a group of toxins, including Ses, which had already been studied for many years [59,60].

The superantigenic activity of staphylococcal enterotoxins is considered one of the main pathogenic virulence factors produced by *S. aureus*. In this context, several genomic sequencing and molecular characterization studies were carried out on *S. aureus* strains isolated from various SFP cases, clinical cases, foods, and animals, thus far registering 33 enterotoxins, including the nine variants discovered in recent years [17,18]. The classic SEs (SEA—SEE), expressed by genes *sea*, *seb*, *sec*, *sed*, and *see*, are the most frequently identified in strains of *S. aureus* associated with outbreaks of food poisoning, the most common being *sea* [4,8]. Previously, it was believed that only classic SEs were responsible for food poisoning. However, recent studies have reported that both classic SEs and SEls are capable of inducing food poisoning and being present in the same *S. aureus* strain [61]. SEG and SEI can stimulate T cell proliferation and elicit emetic responses in the body, but SEI is less stable and requires greater quantities to induce an emetic response [28]. In Switzerland, outbreaks of food poisoning were reported in 2007 and 2014 due to the consumption of goat cheese. Both outbreaks were caused by strains of *S. aureus* that carried the SEls genes *seg*, *sei*, *selm*, *seln*, *sleo*, and *selu*, strains that qualified as CC9 and are frequently isolated in cases of caprine mastitis [62]. The enterotoxins SElS and SElT present in the *pF5* plasmid also exhibited emetic activity. In a study carried out in monkeys, it was reported that SElS and SElT produce emetic activity after 5 and 24 h of gastric administration of 100 µg/kg, respectively [63]. In a study conducted in monkeys, the emetic potential of SEls (SElK, SElL, SElM, SElN, SElO, SElP, and SElQ) was found to be significantly lower than that of SEA and SEB. However, these SEls can still cause SFP in humans [61]. Furthermore, in May 2016, an SPF caused by *S. aureus* harboring the genes *seg*, *sei*, *sem*, *sen*, *seo*, and *selu* was recorded without the production of classic SEs; these findings suggest that SEls (SEG, SEI, SElM, SElN, SelO, and SElU) are also considered as a potential cause of food poisoning outbreaks [15].

In regard to the clinical cases, they observed that enterotoxigenic *S. aureus*, in addition to causing food poisoning, stimulated afferent neurons or induced the release of neurotransmitters from enterochromaffin cells that resulted in vomiting, diarrhea, or intestinal inflammation, which was caused by an interaction process with the enteric nervous system [64]. The virulence factors produced by *S. aureus* can also invade almost all tissues of the human body, causing skin infections, inflammation, pneumonia, and sepsis, leading to various pathological processes [65,66]. Recent research has also shown that SEA can induce DNA damage and trigger an oxidative stress response in hepatocytes and liver tissues [67]. Furthermore, SEC also plays a role in causing human diseases. The subtypes of SEC are named SEC-1 to 4, with SEC-2 and SEC-3 being the most toxic subtypes associated with human vaginal infections. SEC-4 is associated with purpura fulminans and hemorrhagic pneumonia, while SEC-1 is less commonly encountered [68]. However, there are also SEC variants adapted to ruminants, called SEC-bovine and SEC-ovine. The SEC-bovine acts as an inducer for the production of pro-inflammatory cells that cause tissue damage in the mammary glands of ruminants, causing mastitis, a disease commonly found in dairy herds. However, both subtypes are associated with staphylococcal food poisoning, as they have been isolated from milk and dairy products and are responsible for cases of food poisoning. All of these variants share similar structural features but differ in amino acid sequences [69].

On the other hand, *S. aureus* is one of the main etiological agents of bovine mastitis, harboring a wide variety of toxin genes closely associated with the severity of mastitis. The most common SE genes in *S. aureus* isolated from mastitis cases are *sea*, *seb*, *sec*, *sed*, *seg*, *seh*, *sei*, *selj*, *selk*, *sell*, *selm*, *selu*, *selo*, and *selp* [70]. In studies carried out in China, it was reported that enterotoxin M, produced by a clinical strain, induced the release of pro-inflammatory cytokines from bovine mammary cells, which cause cell necrosis [71]. Previously, SElM had been reported in strains of *S. aureus* isolated from cows with chronic mastitis [72].

The genes responsible for superantigen production are often associated with mobile parts of the genetic material, such as plasmids, prophages, pathogenic genomic islands, vSa genomic islands, or genes near the SCC region related to methicillin resistance. This association may explain the presence of genes for various types of superantigens in *S. aureus* strains found in humans and animals [73,74]. The activation of T cells by SAgs differs from conventional activation, as SAgs activate T cells variably, depending on the beta chain, resulting in a widespread activation of the total T cell population. Conversely, conventional activation occurs when a T cell receptor (TCR) recognizes a peptide-MHC II complex. When the TCR specifically binds to the foreign antigen, it triggers a cascade of signals, resulting in the multiplication and specialization of various T cells [75]. A relevant point to consider is that superantigens can induce T cell anergy, a state of unresponsiveness to stimuli, as an immune evasion tactic by *S. aureus*. Exposure to classical SAgs initially results in rapid T cell expansion, followed by a hyporesponsive state with T cell deletions and energy in the remaining cells. This phenomenon is partly mediated by CD8+ regulatory T cells and may depend on SAgs concentration [76]. Additionally, antigen-presenting cells stimulated by SAgs induce pro-inflammatory responses but also activate co-inhibition mechanisms. For non-classical SAgs, there are no reports of T cell anergy so far; therefore, it is important to consider that there may be differences in the properties and immunological effects among different types of SAgs; generalizing to all SAgs may not be appropriate as different SAgs can have distinct effects [75]. Additional studies have shown other highly relevant activities of SAgs, such as interaction with dendritic cells, promoting the production of pro-inflammatory cytokines [77]; hyperactivation of the inflammatory cascade and induction of Th1 or Th17 responses, resulting in excessive tissue damage that compromises the host’s defenses against bacterial infection and thus allowing bacteria to persist in the body for longer periods [78]. Moreover, they are also able to interact with various cells of the immune system (B cells, NKT cells, MAIT cells, mast cells) and interfere with the function of the complement system and neutrophils.

## 8. Mechanisms of Action of the SEs

*S. aureus* employs an extensive variety of virulence factors, such as toxins, to provoke infections in hosts. It is believed that enterotoxins have been involved in foodborne outbreaks since 1900, but it was only in the 1930s that they firmly established an association between them [3]. Most diseases caused by *S. aureus* in humans are due to the contact of the microorganism with two main barriers of the human body: the mucosal barrier (respiratory, gastrointestinal, and genitourinary) and the cutaneous barrier (skin), where the microorganism can interact with the cells of the immune system [79]. Food poisoning caused by the consumption of food contaminated with SEs secreted by *S. aureus* is a special case of disease; the mechanism of action lies mainly in the interaction of SEs in the intestinal tract [80]. SEs pass through the stomach, resist the proteolytic enzymes of the stomach, and reach the intestinal tract, where the emetic action begins, provoking multiple responses from the immune system, the most common being vomiting [61]. The interaction of enterotoxins with epithelial cells disrupts the integrity of cell membranes, increasing intestinal permeability and contributing to gastrointestinal symptoms. Even at extremely low concentrations, various enterotoxins can disrupt immune system homeostasis and interact with the enteric nervous system, stimulating afferent neurons or inducing the release of neurotransmitters, resulting in vomiting, diarrhea, or intestinal inflammation [64]. These reactions are caused by the release of histamine by the submucosal mast cells of the gastrointestinal tract [81]. The target cells of SEs are mast cells in the intestinal tract, and the neurotransmitter 5-hydroxytryptamine (5-HT), known as serotonin, plays a key role in induced emesis [82]. These SEs stand out for their exceptional efficacy in activating T cells, presenting distinctions compared with conventional peptide antigens. While antigen-presenting cells commonly consume and process traditional antigens, SEs follow a unique mechanism for T-cell activation [83].

**Figure 2:**
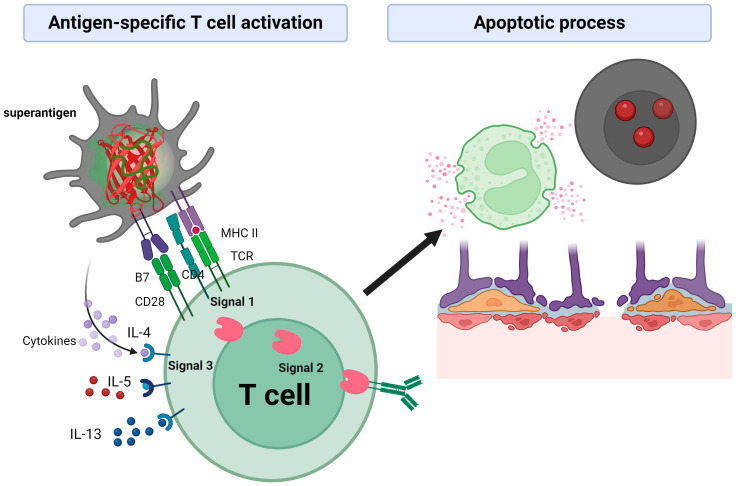
SE superantigens bind directly to the β chain of the TCR (T-cell receptor) molecule, interacting with T-cell scaffold regions and with MHC II (Signal 1) on the surface of APCs (antigen-presenting cells), resulting in the activation of polyclonal T cells (Signal 2) and overproduction of T-cell cytokines, including IL-4, IL-5 and IL-13 (Signal 3); inducing changes in cellular complexity through the apoptotic process. Created with BioRender.com.

SEs are considered superantigens because of their ability to inhibit the immune response, block and destroy B and T cells in the phagocytosis process, and manipulate the innate and adaptive responses of the host immune system [84]. In the case of airway infection, superantigen SEs bind directly to the β-domain variable of the TCR (T-cell receptor) molecule β (β TCRV), interacting with the regions of the T cell scaffold and MHC (major histocompatibility complex) class II on the surface of APCs (antigen-presenting cells). This results in activation of polyclonal T cells and overproduction of T cell cytokines, including IL-4, IL-5, and IL-13 (Figure 2). IL-5 is responsible for eosinophilic inflammation, while IL-4 and IL-13 induce B-cell activation and class change to immunoglobulin E (IgE), promoting local polyclonal IgE response and increased total IgE serum levels [85].

Although SEs have general mechanisms of action, there may be individual variations beyond the specific characteristics of each host type that influence the severity of the response, making some hosts more susceptible or resistant to the effects of enterotoxins. Although animal models may not exactly replicate the human immune response, they demonstrate similar effects of superantigenicity with nonspecific activation of the immune system. There remains a significant limitation in animal model studies due to various issues, including cost, animal availability, ethical considerations, such as in the case of monkeys [86], and in other cases, due to being less susceptible or showing a low response to SEs such as in rats, rabbits, pigs, and mice [78]. However, only enterotoxins that have shown the ability to induce vomiting in monkeys have been designated as “SE”. Those that have not demonstrated such capacity or have not yet been evaluated in non-human vomiting models are termed enterotoxin-like toxins (SEl-) [87].

The research developed by Purwanasari et al. [88] reports that enterotoxin B increases the number of leukocytes, lymphocytes, monocytes, and eosinophils after a 6-day induced infection in mice. However, in the research of Uzunçayır et al. [89], enterotoxin A has been shown to bind to cytokine signaling receptor glycoprotein 130 in humans but does not bind to glycoprotein 130 in mice and rats [89].

SEs are capable of activating a large number of T cells. This activation occurs depending on the Vβ elements in the beta chain of the T cell receptor (TCR) and is directly associated with major histocompatibility complex (MHC) class II molecules on antigen-presenting cells (APCs). Excessive activation of T cells can intensify immune responses, leading to the production of inflammatory mediators such as pro-inflammatory cytokines (including interleukin-2 (IL-2)), tumor necrosis factor-alpha (TNF-α), and interferon-gamma (IFN-γ). These mediators are associated with the development of inflammation, rashes, fever, organ damage, and toxic shock syndrome, both in humans and animals. In humans, the release of leukotrienes and prostaglandins can exacerbate symptoms of staphylococcal food poisoning [83]. As indicated by Hu et al. [86], the fundamentals related to the enterotoxic and emetic activities associated with various *S. aureus* enterotoxins are still lacking elucidation. Non-classical enterotoxins (SEG, SEI, SEM, and SEO) are capable of providing cell death, inducing changes in cell complexity through the apoptotic process, and exhibiting a pro-inflammatory cytokine profile characterized by high levels of TNF-a, IL-6, and IL-12 [90]; which are the most important cytokines in inflammation cases. Additionally, SAgs have been responsible for various human infections such as endocarditis, necrotizing pneumonia, and severe toxic shock syndrome. Few advances have been reported in this area over the years [64,86], and thus, further investigations are needed to identify specific membrane receptors, as well as to understand the mechanisms and structures involved in toxin targeting throughout the nervous system. The strains of *S. aureus* that produce SAgs stand out as relevant etiological agents in various animal diseases. This includes cases of mastitis, both in its clinical and subclinical form, in cattle, goats, sheep, and pigs [91,92,93], as well as occurrences of sepsis, abscess formation, and cutaneous edema in pigs. They have also been associated with lethality in rabbits, arthritis development, and septicemia incidence in birds [94]. It is important to note that the mechanisms of action may vary among different bacterial strains and types of enterotoxins. In the context of mastitis in cattle, it is suggested that superantigenic activity may contribute to bacterial persistence. However, over the decades, efforts have been made to clarify the relationship between enterotoxins and disease severity. It is already known that there are differences in the genetic profile of enterotoxins associated with clinical and subclinical mastitis [95,96], and other studies are beginning to show the role of each in infection. For example, in the case of SEM and SHE, they are responsible for necrosis and apoptosis of bovine epithelial cells [71,97]. According to Fang et al. [98], the authors reported for the first time that SEC can directly induce mastitis in an animal model through superantigenic activity, triggering the release of pro-inflammatory cytokines, inflammatory responses, and, consequently, damage to mammary tissue. In a recent study conducted by Wilson et al. [99], a comprehensive analysis of the *S. aureus* genome was performed to identify present SAgs and the capacity of each toxin to activate specific bovine T cells (bovVβ). The results highlighted that various SAgs demonstrate host-specific functional activity, suggesting that bovine *S. aureus* is capable of efficiently activating the full repertoire of bovine T cells. This finding points to a crucial role in immune system evasion, highlighting the importance of these superantigens in the development of intra-mammary infections. The study’s conclusions emphasized the relevance of the interaction between SAgs and the bovine immune system, with significant implications for a deeper understanding of intra-mammary infection in this specific context. In the same study, the authors did not find the presence of *selt* and *sels* genes in any of the examined genomes, suggesting that these genes are not important in bovine pathogenesis.

A notable characteristic of enterotoxins is their specificity for different animal species. Each enterotoxin variant may show a preference for specific receptors in certain species, such as cattle, sheep, or goats. This specificity is related to the enterotoxin’s ability to bind to specific receptors on the T cells of these animals [69]. Additionally, variation in the immune response triggered by different enterotoxin variants is a critical aspect. The intensity of the inflammatory response, T cell proliferation, and release of pro-inflammatory cytokines may vary among variants.

Deringer et al. [100] investigated the impact of variations of staphylococcal enterotoxin type C (SEC), termed bovine SEC and ovine SEC, on T cells from bovine and human donors. The animal variants of SEC are similar to human-associated SEC (SEC1) but have specific molecular differences for their host species. The researchers observed that all three toxins expanded human T cells carrying certain elements of the T cell receptor. However, SEC1 had a more pronounced effect on one of these elements (huVb12) compared with the bovine and ovine variants. Additionally, bovine T cells responded in a Vβ element-dependent manner to these superantigens. All three toxins induced the proliferation of bovine T cells carrying a specific Vβ element, and SEC1 and ovine SEC were able to stimulate a second element that bovine SEC could not activate. Consequently, these toxins provoke diverse effects on T cells from various animal species, indicating a potential for evolutionary adaptation of the pathogen to influence the immune system in a species-specific manner. These variations in immune response may have a direct impact on the severity of symptoms observed during an *S. aureus* infection. Diversity in immune responses among species, combined with the ability of some variants to trigger more intense responses, may enhance the virulence and persistence of the pathogen in distinct host environments.

## 9. Production of Enterotoxins in Different Environments

*S. aureus* is a pathogen capable of developing in different environments, needing only the necessary conditions for growth and subsequent production of enterotoxins. According to some studies carried out on foods, *S. aureus* can produce heat-stable exotoxins when the bacterial density reaches 10^5^ CFU/g of food [101]. The thermal stability of SEs is influenced by the nature of the food, pH, presence of NaCl, and type of toxin. SEA, for example, is relatively more heat stable at pH 6.0 or higher than at pH 4.5–5.5; SED is relatively more stable at pH 4.5–5.5 than at pH 6, 0, or higher [10].

Some strains of *S. aureus* can produce various amounts of SEs within a period of 24 to 48 h, for example, SEA from 64.45 to 345.02 ng/mL and SEB 2871.28–14,739.17 ng/µL, being 2.4 and 4.6 times greater in 24 and 48 h, respectively [33]. In a study of eggshells, the production of enterotoxin SEA was demonstrated at three different temperatures (10 °C, 25 °C and 37 °C), with a production of 9 lg CFU/g at 25 and 37 °C after 1 day of incubation, but is slower at 10 °C, recording 8.3 lg CFU/g after 5 days of cultivation [102]. The production of SED and SER was demonstrated in meat (666–1225 µg/mL) and milk (54–98 ng/mL) after 24 and 48 h, with SER production being higher in meat juice [103]. In a study conducted with meat products, they observed that the production and expression of SEC enterotoxins were dependent on the fat content and pH of the meat product, with the highest level of SEC expression being observed in chicken and pork ham when inoculating 5 log CFU/cm^2^ [104]. Extended expression of staphylococcal enterotoxin D in ham products was also evaluated, reporting a low but sufficient expression to cause SFP [105].

In seafood, such as fish, strains of *S. aureus* have also been found with multiple genes responsible for producing SEs (SEA, SEB, SEC, SED, SEE, SEG, SEH, SEI, and SEJ). Each gene produces a different type of enterotoxin with similar functional and structural properties [106]. Furthermore, in poultry farms located in Algeria, a high prevalence of MRSA has been reported in turkeys, breeding hens, layers, and broilers; it is more prevalent in turkey farms [107].

On the other hand, *S. aureus* is also a pathogen that colonizes dairy ruminants, such as cattle, buffalo, goats, and sheep, and is the main cause of intra-mammary inflammation (clinical and subclinical mastitis) [108]. Enterotoxin-producing *S. aureus* has also been reported to form part of the skin, mucous membranes, and feces of patients with clinical cases, according to the research developed by [109]. These are subsequently transmitted to food such as milk, cheese, cakes, meats, and foods that have the necessary nutrients for the development of enterotoxigenic *S. aureus* and subsequent expression of enterotoxins.

## 10. Methods for Detecting Staphylococcal Enterotoxins

Due to the significant importance of enterotoxins produced by *S. aureus* in major foodborne outbreaks worldwide, rapid detection is crucial for food safety and public health. There were only five SE identification standards (SEA, SEB, SEC, SED, and SEE) commercially available [13]. Over the years, various methods have been developed, and some can be used for enterotoxin detection; however, it is necessary to evaluate which one is most suitable in terms of efficiency, cost, and speed of results.

### 10.1. Bioassay Method

Bioassays were the first tests developed to confirm the presence of staphylococcal enterotoxins. These assays are also used to determine the impact of various treatments on enterotoxin activity, but they are not the most suitable for routine identification of enterotoxins in foodborne outbreaks because of several disadvantages. These include the cost of maintaining animals, ethical issues related to animal welfare, development of resistance after successive administrations, and lack of specificity [110]. Another drawback is its low sensitivity in experiments, as symptoms of staphylococcal intoxication in animals occur only with doses exceeding 200 ng, which is a high value compared with concentrations found in foods associated with food poisoning outbreaks [2].

### 10.2. Molecular Methods

Polymerase chain reaction (PCR), whole genome sequencing, and, more recently, the Nice automatic Research of alleles (NAuRA) are highly reliable methods employed in the detection of genes associated with enterotoxins [29,111]. The use of molecular techniques for the diagnosis of SEs provides a better understanding of the occurrence, spread, and eradication of staphylococcal enterotoxins. However, these techniques cannot quantify toxin expression. To address this limitation, the use of quantitative polymerase chain reaction (qPCR) is a viable alternative [104,112]. Other techniques, such as oligonucleotide probes [113] and loop-mediated isothermal amplification (LAMP) [114], have been used for the identification of enterotoxin genes. However, it is worth noting that regardless of the chosen molecular approach, initially isolating the pathogen present in the food is necessary, which prolongs the time to obtain the desired results.

### 10.3. Polymer-Based Biosensors

The use of aptamer-based biosensors or polymers for the identification of staphylococcal toxins represents a promising trend in the field of pathogen and toxin diagnostics and detection. These are generally sensitive and specific devices that can be designed to rapidly and accurately detect the presence of staphylococcal toxins [115,116]. These devices use biological macromolecules, such as specific antibodies for the target toxins, such as sensing elements. When the toxins are present, a specific interaction occurs that generates a signal that can be visualized [117]. Polymer-based biosensors are organic macromolecules composed of repetitive units with diverse structures and compositions. Their application includes supporting the immobilization of recognition elements or conferring specific characteristics to the surface. Gupta et al. [118] employed a biosensor with molecularly imprinted polymers (MIPs) for the detection of SEB and suggested their use for constructing sensors with high efficiency for other molecules.

### 10.4. Detecting SEs with Aptamers

Aptamers are oligonucleotides (RNA or DNA) or peptides that are selected for their ability to bind to specific targets with high affinity and selectivity. They are smaller molecules compared with many polymers. Aptamer selection typically involves bioprospecting processes and directed evolution techniques such as SELEX (Systematic Evolution of Ligands by Exponential Enrichment) [119]. Studies have already demonstrated the efficiency of aptamer identification for some toxins such as SEA, SEB, and SEC [120,121,122]), showing great potential for application in food analysis [123]. The detection method can be based on fluorescence, colorimetry, and absorbance [70,120,124]. However, despite being a trend, the practical application of aptasensors for detecting *S. aureus* toxins still presents some challenges, including issues of selectivity and operation that depend on sample conditions (temperature, pH, and viscosity). Other factors, such as nonspecific interactions of the aptamer with the sample matrix and evaluation of the aptasensors only in buffer solutions without testing on real samples, may lead to inaccurate results [117].

### 10.5. Optimum Sensitivity Plate (OSP)

The first immunological method developed for the detection of enterotoxins was the OSP, introduced in the 1950s by Bergdoll [20]. However, this method proved to be insensitive for identifying enterotoxins in food samples and was mainly used to assess the enterotoxigenic potential of staphylococcal strains. From this macro, other techniques, such as micro-immunodiffusion on slides [23] and radioimmunoassay (RIA) [125,126], were developed to enhance the detection capability of enterotoxins at low concentrations. In 1988, Fujikawa [127] and Igarashi introduced Reserve Passive Latex Agglutination (a semi-quantitative test) for the detection of classical enterotoxins (SEA-SEE), and subsequently, being widely commercialized due to their simplicity and rapidity.

### 10.6. Enzyme-Linked Immunosorbent Assay (ELISA)

ELISA is the most widely used method today because of its sensitivity, accuracy, and ease of execution in detecting enterotoxins in food or clinical samples. Its adaptability to different types of samples allows for rapid and quantitative detection [128].

Although there are still few studies focusing on the detection of “non-classical” enterotoxins, internal immunoanalytical methods have been described and are playing a crucial role. Among these is sandwich ELISA, which uses rabbit polyclonal antibodies and has been widely explored in the literature. This approach has been efficient in detecting various enterotoxins, with its application notable for SEG, SEH, SEI [63], and SEIJ [129]. To enhance the specificity of these methods, recent advancements have incorporated at least one murine monoclonal antibody into sandwich ELISA. This notable refinement has been successfully applied to more specific detection of enterotoxins, including SEH [130] and SEI [131], representing a significant evolution in diagnostic accuracy.

### 10.7. Hydrogel-Based Immunobiochip

Aiming for a more comprehensive approach, a hydrogel-based immuno-biochip multiplex prototype was developed. This innovative system demonstrated the ability to simultaneously detect seven enterotoxins, including SEG and SEI [132]. According to Tarisse et al. [110], the immunoassay test developed by their group may represent reliable detection tools for routine investigations of SEA, SEG, SEH, and SEI in both monoplex and multiplex assays. However, the lack of ELISA kits for all currently identified SAgs represents a significant obstacle, preventing characterization of the SAg profile in *S. aureus* strains.

### 10.8. Liquid Chromatography Coupled with High-Resolution Mass Spectrometry (HRMS-LC)

HRMS-LC has been proposed as a relevant complementary method for detecting a limited number of enterotoxins. Lefebvre and colleagues developed a highly specific and sensitive method for the multiplex detection of 24 types of SEs in a culture medium using this technique. To increase the method’s precision, a database of 93 signature peptides was optimized using the complete sequences of the 24 SEs, including their 162 variants. The protocol, based on acid precipitation and enzymatic digestion followed by the detection of specific peptides, has the advantage of simultaneously measuring a large group of toxins. However, due to its laborious nature and limitation in covering all existing SAgs, its feasibility for routine use is not yet established [13]. These multiplex approaches not only expand the variety of detectable toxins but also highlight the feasibility of the practical application of these new technologies.

Overall, this continuous improvement of internal methods not only increases our understanding of the complexities of bacterial toxins but also offers more advanced tools for accurate detection in various situations, ranging from clinical analyses to food assessments.

## 11. Recent Biocontrol Methods against Enterotoxins

The presence of enterotoxins produced by *S. aureus* represents a crucial challenge in the food industry. Therefore, there is an intense search for biological control methods to reduce the presence of these toxins in food. Although industries already implement rigorous Good Manufacturing Practices (GMP) programs and continuous monitoring to prevent bacterial multiplication and toxin formation, it is clear that innovative approaches are needed. These approaches should be comprehensively adapted to the specific characteristics of each food industry and type of food, with the aim of maximizing effectiveness in preventing contamination in both the environment and food products.

A highly promising new approach is the use of beneficial probiotic cultures that have the potential to inhibit *S. aureus* from colonizing and producing toxins [133]. The inhibition of growth by lactic acid bacteria (LABs) results from the production of various compounds, including acids and bacteriocins (identified as the predominant factor influencing both bacterial growth and SE production), along with changes in redox potential associated with environmental stressors. A notable mechanism of action is the ability of organic acids to interact with cell walls and cytoplasmic membranes, resulting in the elimination of pathogens present in food [134]. Pato et al. [135] identified 12 LABs isolated from a type of fermented milk and demonstrated their ability to suppress the growth of *S. aureus*, mainly through the production of organic acids, especially lactic acid, and bacteriocins. In other studies, the expression of SE genes (*sea*, *seb*, *sec*, *seg*, *seh*, *sei*, *selk*, *sell*, *selo*, and *selm*) was strongly inhibited in co-cultures of milk and cheese [136] with different strains of lactic acid bacteria. These studies show a promising path to be explored for the development of new strategies aimed at improving food safety and avoiding the risk of contamination by toxins produced by *S. aureus*.

The application of endolysins in the food industry has also emerged as a promising strategy for the control and reduction of bacterial contamination, notably by pathogenic strains [137]. This method may stand out as an effective approach for the biocontrol of enterotoxins, with several studies already conducted in food systems to control *S. aureus* [138,139]. However, it is relevant to highlight a critical gap in existing research. So far, there have been no systematic evaluations regarding the potential of endolysins to inhibit genes that regulate the production of enterotoxins in food. This observation underscores the pressing need for more in-depth investigations in this specific area. We propose, therefore, a suggestion for future studies to fill this knowledge gap. We consider it crucial to evaluate the potential of endolysins, not only in reducing bacterial load but also in modulating genes that orchestrate the production of enterotoxins. Such research will significantly contribute to understanding the effectiveness of endolysins as a comprehensive tool in the control of foodborne pathogens. We believe that advancements in this area of research will not only promote safer food practices but also open doors for broader and more innovative applications in the biotechnological and medical contexts.

## 12. Conclusions

SEs are structurally homologous with similar nucleotide and amino acid sequences, regulated by various genes found in the genetic structure of *S. aureus*. However, gene regulation and expression are still intricate processes, predominantly at the transcriptional level, and coordinated by various regulatory factors that form complex networks. Around these, various identification methods were developed, such as Polymerase Chain Reaction (PCR), whole genome sequencing, and most recently, the Nice automatic Research of alleles (NAuRA). SEs were identified in various food poisoning outbreaks, mainly related to dairy products and derivatives, meats, prepared products, and utensils. The presence of SEs produced by *S. aureus* represents a crucial challenge in the food industry. Therefore, there is an intense search for biological control methods to reduce and control the presence of these toxins, and this implies from rigorous methods of good manufacturing practices to new methods with the potential to inhibit the growth of enterotoxigenic *S. aureus*.

## Data Availability

No new data were created or analyzed in this study. Data sharing is not applicable to this article.
